# Benign Symmetric Lipomatosis of the Tongue With Dysgeusia: A Case Report and Literature Review

**DOI:** 10.7759/cureus.76779

**Published:** 2025-01-02

**Authors:** Hiroshi Hyakusoku, Yoshiaki Mori, Mayumi Yakeishi, Risa Kamoshida, Meijin Nakayama

**Affiliations:** 1 Otolaryngology, Yokosuka Kyosai Hospital, Yokosuka, JPN; 2 Pathology, Yokosuka Kyosai Hospital, Yokosuka, JPN

**Keywords:** benign symmetric lipomatosis, dysgeusia, madelung disease, multiple symmetric lipomatosis, symmetric lipomatosis of the tongue

## Abstract

Benign symmetric lipomatosis (BSL) is a rare disorder that is characterized by diffuse growth, multiple symmetrical accumulation, and unencapsulated lipomas. BSL is also known as Madelung disease, Launois-Bensaude syndrome, and multiple symmetric lipomatosis. BSL is commonly found in the upper trunk and posterior neck. Symmetric lipomatosis of the tongue (SLT) is extremely rare. A 49-year-old man with a history of primary biliary cholangitis, hypertension, and gastroduodenal ulcer, as well as a background of heavy alcohol consumption, was referred to our department due to a one-month history of difficulties in speaking and swallowing and dysgeusia. Bilateral adipose tissue in the tongue was observed. His dysgeusia improved immediately after glossectomy. A literature review of case reports of SLT was performed using PubMed and Web of Science. A total of 62 articles were extracted. We reviewed 17 articles comprising 18 patients (15 men and three women) who met the inclusion criteria. Including our patient, the age at diagnosis ranged from 49 to 88 years old, with a median age of 66.5 years old. The rates of alcohol consumption and type of BSL (I/II) were 42.1% and 84.2%, respectively. The rates of dysphasia, dysarthria, and dyspnea symptoms were 55.6%, 66.7%, and 27.8%, respectively. Fourteen patients underwent glossectomy. Dysgeusia caused by SLT may improve with glossectomy. ‘Wait-and-see’ may be an option unless patients complain of symptoms such as dysphasia, dysarthria, and dyspnea. As lipomatosis has the potential for regrowth and SLT can transform into a malignant tumor, long-term follow-up is necessary.

## Introduction

Benign symmetric lipomatosis (BSL) is a rare disease, characterized by diffuse growth, multiple symmetrical accumulation, and unencapsulated lipomas [1]. BSL is also known as Madelung disease, Launois-Bensaude syndrome, and multiple symmetric lipomatosis. Otto Madelung described the classic house collar cervical distribution of the lipomatous tissue, while Launois and Bensaude further defined the syndrome by the presence of multiple symmetric unencapsulated fatty accumulations [2,3]. Symmetric lipomatosis of the tongue (SLT) is extremely rare and was first reported by Desmond in 1944, which is relatively recent compared to BSL, initially described by Brodie in 1846, with Madelung later reporting 35 patients with cervical lipomatosis [3-5]. Patients with SLT often present with symptoms such as dysphagia, dysarthria, and dyspnea, which can typically be managed with surgical resection [5-7]. Given that BSL predominantly affects the upper trunk and posterior neck, it remains controversial whether BSL and SLT should be categorized as the same disease entity. Herein, we report a case of SLT presenting with dysgeusia and provide a literature review of the clinical characteristics.

## Case presentation

**Figure 1:**
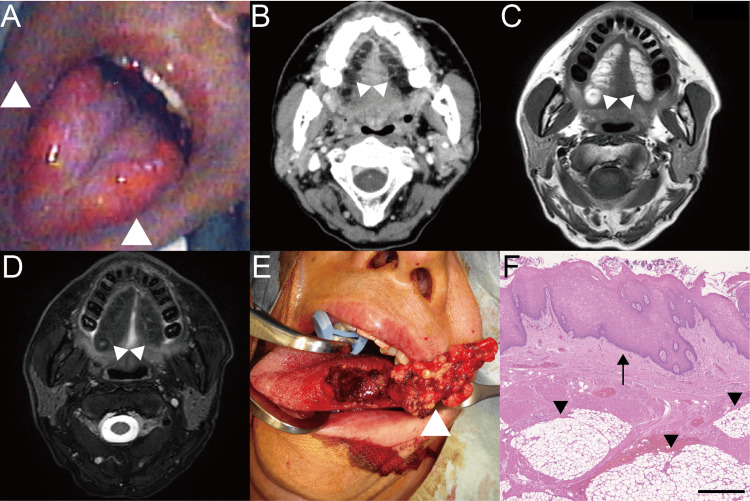
Images of the present case (A) Yellowish tumors in the right and left edges of the tongue (arrowheads); (B) Bilateral adipose tissue in the tongue on CT (arrowheads). The right side was 39.0 x 9.5 mm and the left side was 31.2 x 11.9 mm; MRI reveals adipose tissue with a hyperintense signal on T1-weighted imaging and a hypointense signal on T2-weighted imaging (C: T1-weighted, D: T2-weighted, arrowheads); (E) Unencapsulated adipose tissue invading the lingual muscle (arrowhead); (F) Unencapsulated mature adipose tissue is found (arrowhead). No taste buds were found in the epithelium (arrow). Hematoxylin and eosin stain. Scale bar: 500 μm. CT, Computed tomography; MRI, Magnetic resonance imaging

A 49-year-old man with primary biliary cholangitis underwent treatment from the gastroenterologist at our hospital. He also had hypertension and a gastroduodenal ulcer. His medical history included heavy alcohol consumption and 20 cigarettes per day since he was 18 years old. He was referred to us with a one-month history of difficulties in speaking and swallowing, as well as dysgeusia. Mild indurations in the bilateral tongue were observed (Figure 1A). A biopsy was performed on the mucosa and submucosa on the left side of the tongue under local anesthesia, revealing adipose tissue and atypical squamous epithelium. No abnormal findings were found in the blood tests. Computed tomography revealed adipose tissue in the bilateral tongue (Figure 1B), but no lipomatous tumors in his neck or trunk. Magnetic resonance imaging (MRI) revealed a lesion composed of adipose tissue with a hyperintense signal on T1-weighted imaging and a hypointense signal on T2-weighted imaging (Figures 1C, 1D). Zinc and copper levels in laboratory chemistry (88 μg/dL and 128 μg/dL, respectively) were within normal ranges. Electrogustometry showed no responses at the bilateral glossopharyngeal nerves or chorda tympani nerve areas in the tongue. We diagnosed SLT with dysgeusia. As his dysgeusia did not improve after one month of oral zinc acetate dehydrate at a dose of 25 mg twice a day, and to also address his speaking and swallowing difficulties, we performed lateral glossectomy twice under general anesthesia (Figure 1E). Because dysgeusia improved on the left side immediately after the left glossectomy, the right glossectomy was performed two months later to improve dysgeusia on the right side, and because the patient still complained of dysarthria and dysphagia. Four months after the second surgery, the electrogustometry threshold was 8 dB on the right and 6 dB on the left in the chorda tympani nerve areas of the tongue. However, no responses were observed in the bilateral glossopharyngeal nerve areas. Histologic examination revealed mature lipocytes with fibrous septa incorporating skeletal muscle and no taste buds in the epithelium (Figure 1F). Both pathological results were identical. We diagnosed lipomatosis based on the absence of malignant tumors and the lack of encapsulated fatty tissue characteristic of lipomas. The patient had no complaints of dysphagia, dysarthria, or dysgeusia. One year after surgery, there was no recurrence in his tongue.

Methods

A literature review of case reports of SLT was conducted on PubMed and Web of Science using the keywords 'Madelung disease and tongue' and 'benign symmetric lipomatosis (BSL) and tongue'. All publications retrieved up to November 11th, 2023, were screened for relevance based on titles and abstracts. The inclusion criteria were full-text articles written in English. The exclusion criteria were full-text articles written in languages other than English, cases of Madelung disease or BSL without evidence of SLT, diagnosed as lipoma, or conference proceedings.

Results

**Figure 2:**
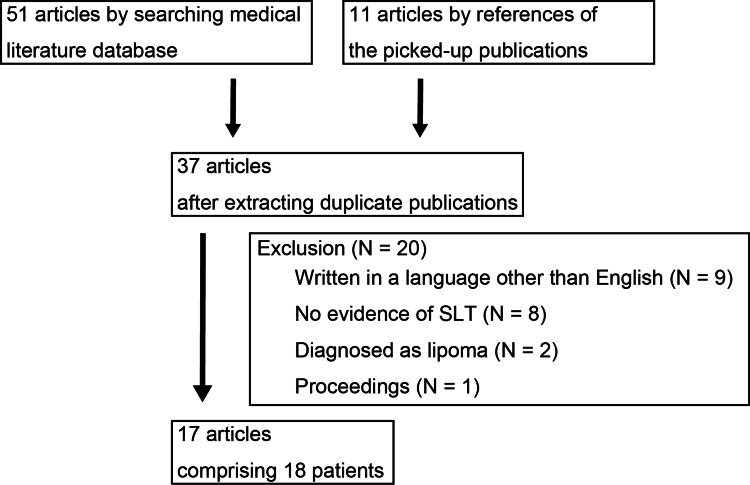
Flow diagram for the literature search SLT, Symmetric lipomatosis of the tongue

A total of 62 articles (51 by searching the medical literature database and 11 extracted from the references of the extracted publications) were initially identified. After excluding duplicate publications, 37 articles were selected. Articles were further excluded according to the exclusion criteria: full-text articles written in a language other than English (N = 9), cases of Madelung disease or BSL without evidence of SLT (N = 8), diagnosed as lipoma (N = 2), or conference proceedings (N = 1). We reviewed 17 articles comprising 18 patients (15 men and three women) who met the inclusion criteria (Figure 2).

Clinical characteristics

Clinical characteristics of the case reports and the present case are shown in Table 1 [1,2,5-19]. The BSL type was analyzed using the classification of Schiltz et al. (see Table 2) [20]. The age at diagnosis ranged from 49 to 88 years old, with a median age of 66.5 years old. The rates of alcohol consumption were 42.1%. Two cases were type I (10.5%), two cases were type III (10.5%), and 15 cases involved only the tongue (78.9%). The cohort consisted of 11 East Asians and five patients of Mediterranean descent. The rates of dysphasia, dysarthria, and dyspnea (Y/N) symptoms were 61.1%, 66.7%, and 27.8%, respectively. Fourteen patients underwent glossectomy.

**Table 1:** Clinical characteristics of patients with benign symmetric lipomatosis of the tongue BPG, Bilateral partial glossectomy; COPD, Chronic obstructive pulmonary disease; DM, Diabetes mellitus; F, Female; GERD, Gastroesophageal reflux disease; HT, Hypertension; LPG, lateral partial glossectomy; M, Male; N, No; OT, Only the tongue; Y, Yes

Author	Year	Age	Sex	Alcohol consumption	BSL type	Country	Dysphasia	Dysarthria	Dyspnea	Surgical treatment	Outcome	Systemic disease
Desmond [5]	1947	67	M	N	OT	United Kingdom	Y	Y	N	Left LPG	Improved dysphasia and dysarthria	DM, stroke, mentally defective
Ogawa et al. [8]	1988	67	M	Y	OT	Japan	Not clear	Not clear	Not clear	No surgeries		Stroke
Katou et al. [9]	1993	61	M	N	OT	Japan	Y	Y	N	BPG	Not clear	Gastric cancer
Katou et al. [9]	1993	71	M	Y	OT	Japan	N	N	N	BPG (two times)	Not clear	HT
Calvo-Garcia et al. [10]	1999	65	M	N	OT	Spain	N	N	Y	No surgeries		-
Vargas-Díez et al. [2]	2000	59	M	N	Ia	Spain	Y	Y	N	BPG	Not clear	-
Jinbu et al. [11]	2004	72	M	N	Ic	Japan	Y	Y	N	Left LPG	Not clear	DM
Lopez-Ceres et al. [12]	2006	57	F	N	III	Spain	Y	Y	Y	BPG	Not clear	HT, colorectal cancer, hyperuricemia, psoriasis
Ishikawa et al. [13]	2006	63	M	Y	OT	Japan	Y	N	N	Left LPG	Not clear	Alcoholic hepatitis, Hyperlipidemia
Ettl et al. [1]	2009	49	M	Y	III	Germany	Y	Y	N	BPG	Not clear	Alcoholic liver cirrhosis, COPD
Murakami et al. [14]	2009	88	M	N	OT	Japan	N	N	N	No surgeries		-
Vasileiadis et al. [6]	2013	67	M	N	OT	Greece	Y	Y	Y	BPG	Improved dysphasia, dysarthria, and dyspnea	DM, COPD
Kang et al. [15]	2013	76	F	N	OT	South Korea	N	N	N	No surgeries		-
Azuma et al. [16]	2015	74	M	N	OT	Japan	N	Y	N	BPG	Not clear	Myocardial infarction
Kudoh et al. [17]	2016	80	M	Y	OT	Japan	Y	Y	N	BPG	Improved dysarthria and dysphasia	alcoholic liver injury, HT, DM, acute pancreatitis, dementia
Yáñez et al. [7]	2018	65	F	N	OT	Spain	Y	Y	Y	BPG (two times)	Improved dysphasia, dysarthria, and dyspnea	Sleep apnea, obesity, HT, cholecystectomy,
Murayama et al. [18]	2020	66	M	Y	OT	Japan	N	Y	N	BPG	Not clear	Alcoholic cirrhosis, DM, megaloblastic anemia, GERD
Bastos et al. [19]	2020	87	M	Y	OT	Brazil	N	N	N	No surgeries		Rheumatoid arthritis
Hyakusoku et al. (present case)	2023	49	M	Y	OT	Japan	Y	Y	N	BPG (two times)	Improved dysphasia, dysarthria, and dysgeusia	Primary biliary cholangitis, HT, gastroduodenal ulcer

**Table 2:** Different phenotypes of the Schiltz classification Source: [20]

Type	Subtype	Distribution
I		Upper body
	Ia	Neck
	Ib	Neck + shoulder girdle + upper arms
	Ic	Neck + shoulder girdle + upper arms + trunk
II		Lower body (hips/buttocks and upper legs)
III		A general distribution, apart from head, forearms, and lower legs

## Discussion

This is the first case report describing a patient who complained of dysgeusia without hypozincemia and recovered after glossectomy. We performed lateral partial glossectomy twice to assess the impact on dysgeusia. The decision for the second surgery was prompted by the immediate improvement in the patient's taste after the initial procedure. Currently, the patient has no complaints of dysphagia, dysarthria, or dysgeusia. Lipomatosis may have affected the taste buds, resulting in dysgeusia.

The tongue senses taste through taste buds, with the anterior two-thirds of the tongue being innervated by the chorda tympani nerve, and the posterior one-third by the glossopharyngeal nerve [21]. The anterior portion of the tongue contains fungiform papillae, while the back contains foliate papillae, and the base contains circumvallate papillae, all of which house taste buds [22]. The site of SLT is located on the anterior two-thirds of the tongue, in the area innervated by the chorda tympani nerve, which includes the taste buds of the fungiform papillae. In this case, despite the absence of lipomatosis in the posterior one-third of the tongue, taste loss was observed in this region regardless of the presence of lipomatosis. During electrogustometry of the anterior two-thirds of the tongue, the probe was placed over the lipomatous tissue. A significant change in threshold values was noted before and after surgery, suggesting that the fungiform papillae were covered by adipose tissue, leading to taste disturbance.

To elucidate the clinical characteristics of SLT, we performed a literature review. SLT has similarities to BSL. Vasileiadis et al. and Azuma et al. [6,16] reported a similar sex ratio with a predominance in men but reported differences in the age of onset, fat deposition in the upper trunk and neck, and patient ethnicity between SLT and BSL. Furthermore, alcohol consumption is associated with BSL, and our findings suggest an association with SLT. The Schiltz classification applies to cases involving regions of the body below the neck [20]. However, when lipomatosis occurs in the tongue, it does not fit into any category. Notably, 78.9% of SLT patients did not exhibit involvement of areas below the neck. Thus, it remains controversial whether BSL and SLT should be categorized as the same disease. As most SLT patients were male and East Asian, there may be a genetic association.

More than half of the patients reported dysphagia and dysarthria attributed to macroglossia, prompting their visits to medical institutions. Dyspnea is less common. The decision for surgery may be triggered by the severity of symptoms, as patients who decline glossectomy typically do not complain of these symptoms. A ‘wait-and-see’ approach may be an option unless the patient complains of severe symptoms.

BSL is strongly associated with alcohol consumption. Li et al. reported that almost 90% of patients consumed alcohol [23]. In our study, 42.1% of patients consumed alcohol, suggesting an association with SLT. Furthermore, when focusing only on Japanese men, 60% had a history of alcohol consumption. When alcohol is oxidized to acetaldehyde, and eventually to acetyl CoA, nicotinamide adenine dinucleotide (NAD) is required to reduce hydrogen atoms and electrons, and NAD^+^ is subsequently reduced to the product NADH + H^+^ [24]. Acetyl CoA is a metabolite produced from all major nutrients including triglycerides and fat. Therefore, excessive alcohol consumption may cause the accumulation of triglycerides, leading to fatty liver and obesity due to the accumulation of fat tissues in the body. Excessive calorie consumption may also be associated with fatty liver and obesity for the same reason. Thus, excessive alcohol consumption may cause BSL, and excessive calories or alcohol consumption may contribute to the development of SLT.

Histopathologic examination is essential for diagnosing SLT. Histologic findings include mature lipocytes with fibrous septa incorporating skeletal muscle and nonencapsulated adipose tissue, differentiating SLT from lipoma [17]. Complete resection is challenging. Furthermore, due to the potential for lipomatosis regrowth and the transformation of SLT into a malignant tumor, though this is very rare and has only been reported in three cases: liposarcoma or intramyxoid sarcoma [25], long-term follow-up is necessary.

## Conclusions

SLT is extremely rare. This is the first case report of a patient who presented with dysgeusia, where taste improved after glossectomy. In the literature review, the median age at diagnosis was 66.5 years old. The majority of patients were male and East Asian. The rate of alcohol consumption was 42.1%, and the rates of dysphasia, dysarthria, and dyspnea symptoms were 55.6%, 66.7%, and 27.8%, respectively. Fourteen patients underwent glossectomy. A ‘wait-and-see’ approach may be an option unless patients complain of severe symptoms. Complete resection is challenging. As lipomatosis has the potential for regrowth and SLT may transform into a malignant tumor, long-term follow-up is necessary.
